# Herpes virus infection associated with interstitial nephritis in a beaked whale (*Mesoplodon densirostris*)

**DOI:** 10.1186/1746-6148-8-243

**Published:** 2012-12-13

**Authors:** Manuel Arbelo, Edwige N Bellière, Eva Sierra, Simona Sacchinni, Fernando Esperón, Marisa Andrada, Miguel Rivero, Josue Diaz-Delgado, Antonio Fernández

**Affiliations:** 1 Unit of Veterinary Histology and Pathology, Institute for Animal Health (IUSA), Veterinary School, University of Las Palmas de Gran Canaria, Canary Islands, Spain; 2 Research Centre for Animal Health (CISA - INIA), Madrid, Spain

**Keywords:** Beaked whale, Interstitial nephritis, Alpha herpes virus, Diagnosis

## Abstract

**Background:**

The capacity for herpesvirus to cause disease in cetaceans is unclear and may be varied depending on the different conditions of individuals and between different species. Kidney pathology and intralesional virus-associated infection have been rarely reported in cetaceans.

**Result:**

On April 2004, an old adult male Blainville’s beaked whale (*Mesoplodon densirostris*) 420 cm long with a poor body condition was stranded on Tenerife Island. During necropsy, no gross lesions were observed in the kidneys. However, membranous glomerulonephritis, multifocal interstitial lymphoplasmacytic nephritis and acute multifocal necrotizing tubulointerstitial nephritis with intranuclear inclusion bodies was diagnosed by histological analysis. Tissue samples were submitted for bacteriological analysis and molecular viral screening.

**Conclusion:**

A novel alpha herpesvirus associated with interstitial nephritis was identified in an old adult male Blainville's beaked whale (*M. densirostris*) with a poor body condition stranded in the Canary Islands. This report suggests that identification of herpesvirus infection could be used as a differential diagnosis for interstitial nephritis in cetaceans.

## Background

The presence of herpesviruses (HV) in cetaceans was shown in the late 1980s by electron microscopy (EM) analysis demonstrating HV-like particles in skin biopsies from beluga whales (*Delphinapterus leucas*) [1,2]. EM analysis also demonstrated that HV was associated with encephalitis in a harbor porpoise (*Phocoena phocoena*) [3] and with skin lesions of dusky dolphins (*Lagenorhynchus obscures*) [4]. In addition to EM, immunoperoxidase staining [3], serum neutralization and enzyme-labeled immunosorbent assays have been used as indicators for the presence of HV in cetaceans [5]. Alpha-HV have been associated with fatal systemic infections in bottlenose dolphins (*Tursiops truncatus*) [6], a Cuvier’s beaked whale (*Ziphius cavirostris*) [7], and with cutaneous lesions in bottlenose dolphins [8]. Gamma-HV have been identified in mucosal lesions in bottlenose dolphins, Risso’s dolphins (*Grampus griseus*), dwarf sperm whales (*Kogia sima*) and Blainville’s beaked whales (*Mesoplodon densirostris*) [8-10].

The capacity of HV to cause disease in cetaceans is unclear and may show variations depending on the different conditions between individuals or within species [7-10]. Recently, systemic herpes viral lesions have been reported in a striped dolphin, likely secondary to the immunosuppression caused by morbillivirus co-infection [11].

Tubulointerstitial nephritis is characterized by inflammatory and degenerative changes in tubular epithelium associated with interstitial lymphoplasmacytic cell infiltration [12]. Interstitial lymphoplasmacytic nephritis is a frequent histopathological finding in cetaceans. However, intralesional etiologies or pathogenic virus or bacteria have rarely been associated with this type of lesion. Here we report the presence of tubulointerstitial nephritis in a beaked whale stranded in the Canary Islands caused by a novel cetacean cytopathogenic alpha-HV.

## Methods

**Figure 1:**
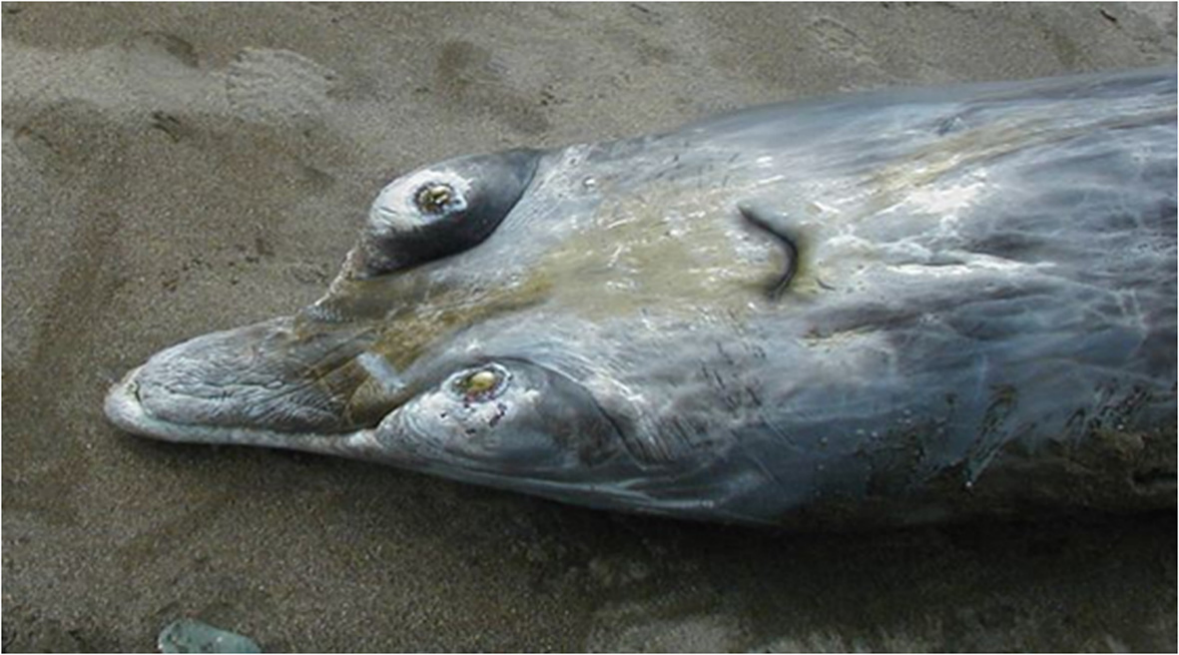
**Stranded specimen involved.** An adult old male Blainville’s beaked whale (*Mesoplodon densirostris*). Mandibule and tooth features match with an old male beaked whale.

On April 18th, 2004, an old adult male *Mesoplodon densirostris* (420 cm long) with a poor body condition was observed to be alive near the shore on Tenerife Island (Granadilla de Abona, Geographic Universal Trasverse Mercator (UTM) coordinate system, X 348338, Y 3102515). The whale died soon after beaching (Figure 1). Necropsy was started 8 hours postmortem (code 1–2) [13].

Routine sampling for histological, EM, immunohistological, bacteriological and virological studies were performed [7]. Samples fixed in 10% neutral buffered formalin were routinely processed and embedded in paraffin blocks. Histological sections were stained using hematoxylin and eosin (H&E) as well as periodic acid Schiff (PAS). Additionally, immunohistochemistry (IHC) for the detection of morbillivirus and HV antigens was performed as previously described [14,15] Tissue samples from lung, liver and kidney were submitted for bacteriological analysis [7,13].

For molecular viral screening, 0.5 g of frozen tissues samples from skin, liver, muscle, lung and kidney were macerated in 4.5 ml of phosphate buffered saline (PBS), using an automatic homogenizer (Precellys® 24 Dual homogenizer, Bertin Technologies, Montigny le Bretonneux, France), and total nucleic acid was extracted by pressure filtration (QuickGene DNA tissue kit S, FujiFilm Life Science, Tokyo, Japan), following the manufacturer’s instructions.

HV DNA detection was conducted by pan nested HV PCR [16] involving DFA, KG1 and ILK primers in the external PCR, and TGV-IYG primers in the internal PCR. The primer sequences have been described previously [16]. To determine positive samples, direct sequencing of the nested PCR product, approximately 220 base pairs (bp) was performed using the primers TGVseq – IYGseq.

To obtain additional nucleotide sequences (approximately 700 bp in length), products of the external PCR from positive samples were amplified using the primers DFA and KG1. Amplified DNA fragments of the predicted size were purified and cloned into the plasmid vector pGEM-T (Promega Corporation) following the manufacturer’s instructions and sequenced with the corresponding T7 primer (forward) and SP6 primer (reverse).

Two clones for each sample were selected. Once the consensus nucleotide sequence was obtained and the primer binding sites excluded, the Clustal W option in MEGA 4.0 was used to deduce the corresponding amino acid sequence and to construct an unrooted neighbor-joining tree. All the available cetacean HV DNA-polymerase gene sequences reported to date [6-11,17-20] were compared by their aminoacid (aa) sequences in the GenBank database, which is the recommended method for molecular HV classification [21].

Sequences that were 100% identical were excluded (in those cases, only the sequence published first was conserved in the phylogenetic tree) and other sequences from HV-assigned species available at the International Committee on Taxonomy of Viruses [22] were also included in the comparison.

## Result

### Pathological findings

Gross examination revealed several *Xenobalanus sp* barnacles attached to the fluke, multiple cutaneous scars and atrophy of the epaxial musculature. Intravascular gas was noted within the epicardial, mesenteric and portal veins as well as beneath the liver capsule. Nematodes were observed within the bronchi. Fish bones, otoliths and plastic foreign debris were found within the squamous stomach. Many parasitic cysts (*Phyllobothrium sp.)* were observed in the retroperitoneal area as well as in testicular serosa. The kidneys were congested and no urine was found in the bladder.

**Figure 2:**
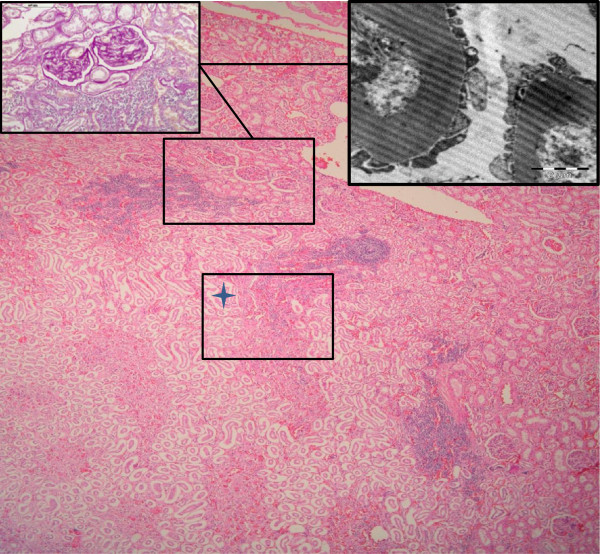
**Histological, histochemical and ultrastructural findings.** Kidney. Cortico- medular area of one reniculum. Multifocal lymphoplamacytic interstitial nephritis. H&E (100X, bar=200 μm). Up right side inset: Higher magnification of renal cortical area showing aggregate of lymphoplasmocytic cells and two glomeruli with (PAS positive). PAS technique (400x, barr= 100 μm). Up right side inset: EM picture showing thickening of glomerular basement membranes. (Bar 2 μm, EM technique).

**Figure 3:**
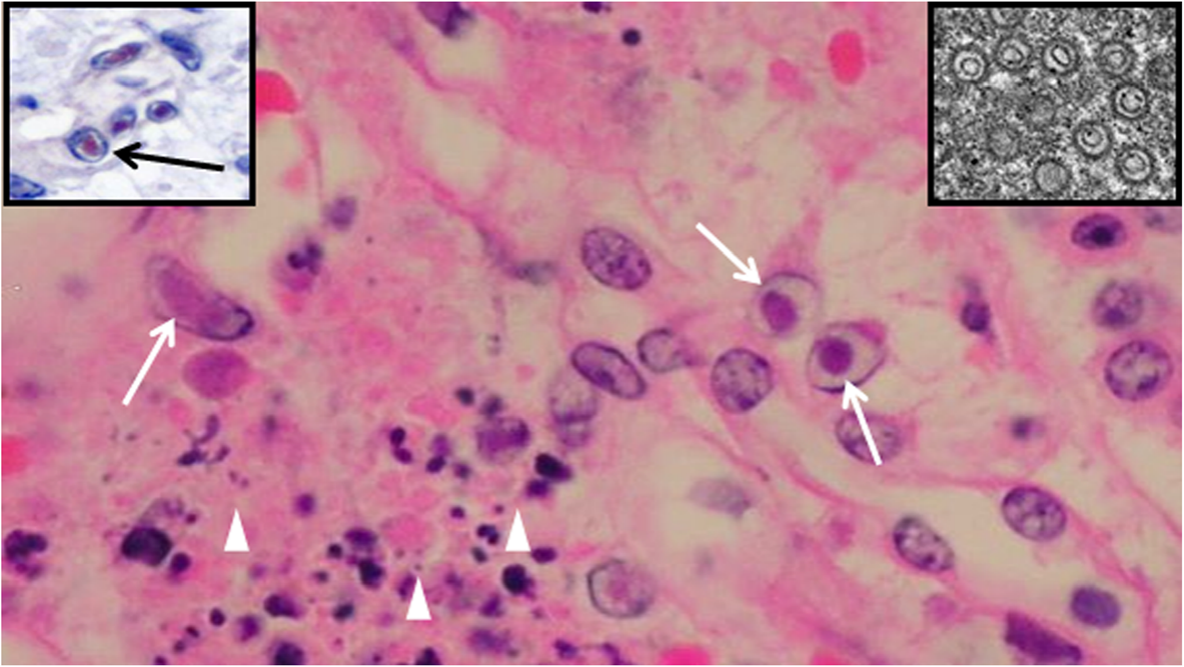
**Immunohistological and ultrastructural interstitial kidney findings.** Kidney. Higher magnification of Figure 2 (box with star). Interstitial cell necrosis (arrowheads) and intranuclear inclusion bodies (arrows) in tubuloepithelial cells. H&E (bar=10 μm). Inset up right: Herpes virus particles (EM) (bar=10 nm). Inset up left: Inclusion bodies labeled immunpositivelly for herpesvirus antigen (red colour) (arrow). IHC counterstaining with hematoxilin (bar=10 μm).

Histologically, membranous glomerulonephritis was the main finding in the glomeruli (Figure 2, insets). A multifocal lymphoplasmacytic interstitial nephritis was present in 33% of the reniculi by microscopic analysis (Figure 2). Multifocal interstitial and tubuloepithelial necrosis with the presence of intranuclear inclusion bodies in tubuloepithelial cells was observed (Figure 3) in the reniculi, which also presented with membranous glomerulonephritis and lymphoplasmacytic interstitial nephritis. Additional findings included intravascular non-staining vacuoles (gas-like bubbles) in the hepatic sinusoids, pericardial vessels, within small vessels of intestinal serosa and inter-renicular venous vessels. Mild multifocal parasitic bronchopneumonia with intraluminal nematodes was noted. Moderate diffuse intracytoplasmic eosinophilic globules were present in the hepatocytes, as frequently seen in active (alive) stranded dolphins (personal observation). Cortico-adrenal steatosis and multifocal lymphoplasmacytic adrenalitis were observed in both adrenal glands but no cell necrosis and/or viral inclusion bodies was found. Signs of testicular atrophy, likely associated with senescence, were observed with increased interstitial connective tissue and thickened basement membrane surrounding the atrophied tubules with no luminal spermatozoa.

### Immunohistological and ultrastructural study

HV antigen was only detected in the kidney and immunopositivity was clearly observed in the intranuclear inclusions bodies of tubuloepithelial cells and small amount was associated with cell debris in necrotic areas. (Figure 3, inset). Ultrastructural analysis demonstrated that intranuclear inclusion bodies labeled immunohistologically corresponded to HV particles (Figure 3, inset).

### Bacteriological analysis

*Photobacterium damsela* and *Pasteurella skyensis* were isolated from lung samples. *Clostridium sp* was isolated from the liver. No bacteria were isolated from kidney samples.

### Molecular evaluation

**Figure 4:**
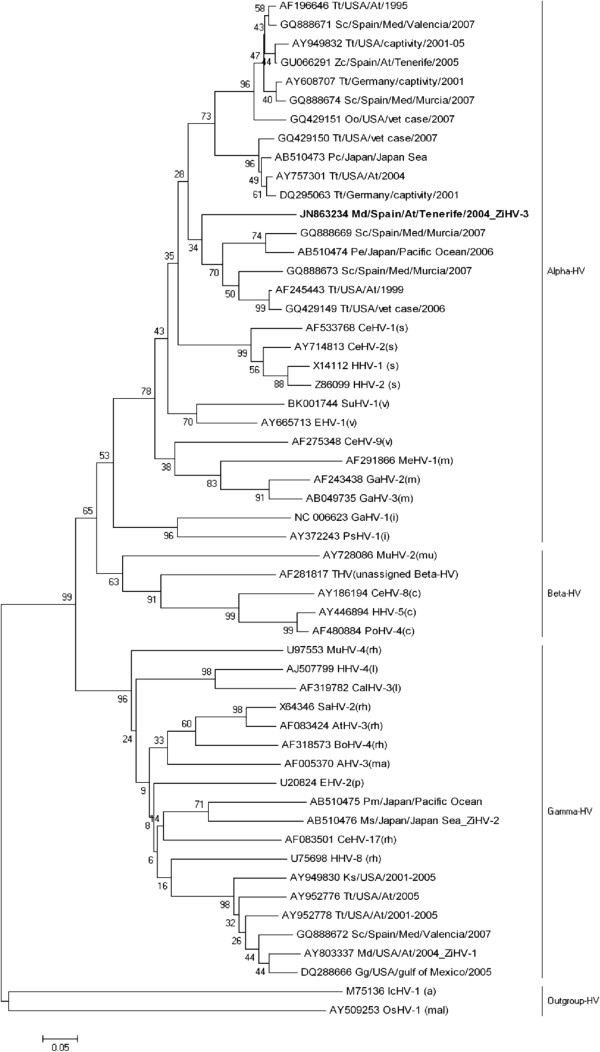
**Phylogenetic analysis.** Phylogeny reconstruction made by the Neighbor-Joining method, with a Bootstrap test of 1000 replicates, and a substitution model based on amino acid (aa) p-distance, inferred above 47 aa and including 54 taxa. In the case of herpesviruses from the ICTV, each sequence name refers to the GenBank accesion number, the virus name and to the Family or Gender it belongs to [Family *Herpesviridae:* Subfamilia *Alphaherpesvirinae:* Gender *Simplexvirus* (s): *Human herpesvirus 1* (HHV-1), *Human herpesvirus 2* (HHV-2), *Cercopithecine herpesvirus 1* (CeHV-1), *Cercopithecine herpesvirus 2* (CeHV-2); Gender *Varicellovirus* (v): *Suid herpesvirus 1* (SuHV-1), *Equine herpesvirus 1* (EHV-1), *Cercopithecine herpesvirus 9* (CeHV-9), Gender *Mardivirus* (m): *Meleagrid herpesvirus 1* (MeHV-1), *Gallid herpesvirus 2* (GaHV-2), *Gallid herpesvirus 3* (GaHV-3); Gender *Iltovirus* (i): *Gallid herpesvirus 1* (GaHV-1), *Psittacid herpesvirus 1* (PsHV-1); Subfamily *Betaherpesvirinae*: Gender *Cytomegalovirus* (c): *Cercopithecine herpesvirus 8* (CeHV-8), *Human herpesvirus 5* (HHV-5), *Pongine herpesvirus 4* (PoHV-4), Gender *Muromegalovirus* (mu): *Murid herpesvirus 2* (MuHV-2), unassigned Beta-HV: *Tupaiid herpesvirus 1* (THV); Subfamily *Gammaherpesvirinae*: Gender *Lymphocryptovirus* (l): *Human herpesvirus 4* (HHV-4), *Callitrichine herpesvirus 3* (CalHV-3); Gender *Rhadinovirus* (rh): *Saimiriine herpesvirus 2* (SaHV-2), *Ateline herpesvirus 3* (AtHV-3), *Bovine herpesvirus 4* (BoHV-4), *Cercopithecine herpesvirus 17* (CeHV-17), *Murid herpesvirus 4* (MuHV-4), *Human herpesvirus 8* (HHV-8), Gender *Percavirus* (p): *Equid herpesvirus 2* (EHV-2), Gender *Macavirus* (ma): *Alcelaphine herpesvirus 1* (AHV-1)]; Family *Alloherpesviridae* (a): *Ictalurid herpesvirus 1* (IcHV-1); Family *Malacoherpesviridae* (mal): *Ostreid herpesvirus 1* (OsHV-1). In the case of the cetacean herpesviruses sequences, names are refering to the GenBank accesion number, the cetacean species [Species: Tt, *Tursiops truncatus*; Sc, *Stenella coeruleoalba*, Zc, *Ziphius cavirostris*; Oo, *Orcinus orca*; Pc, *Pseudorca crassidens*; Pe, *Peponocephala electra*; Pm, *Physeter macrocephalus*; Ms, *Mesoplodon stejnegeri*; Kg, *Kogia sima*; Md, *Mesoplodon densirostris*; Gg, *Grampus griseus*] and the country.

Direct sequencing was successful for the internal PCR product from the kidney sample. However, cloning was necessary for the internal PCR product from the lung sample and for the external PCR product of the kidney sample. The same 181 bp (60 aa) sequence was obtained from kidney and lung samples, and a 692 bp (230 aa) sequence was obtained from the kidney alone (GenBank accession number JN863234). Phylogenetic analysis (Figure 4) demonstrated that the sequence obtained in this study is a novel HV.

## Discussion

Although in this unique case of stranding the cause of death was not determined, two pathological findings merit consideration. First, a systemic gas embolism was observed similar to that linked to military sonar described in stranded beaked whales [13]. However, it has been determined that no anthropogenic sound source was spatially or temporally used in the area of stranding. Thus, we suggest this kind of gas embolism may be linked to diving problems in “sick”, stranded beaked whales (Fernandez, personal communication).

Second, although no gross lesions were observed in the kidneys, two main histopathological findings were noted. Membranous glomerulonephritis, multifocal interstitial aggregate of lymphoplasmacytic cells, multifocal necrotizing tubulointerstitial nephritis and the presence of intranuclear inclusion bodies in epithelial cells were observed.

In terrestrial mammals, interstitial nephritis may be the result of bacterial and viral septicemia, whereby these infectious agents first infect kidney tubules and then induce an inflammatory response in the interstitium [23].

IHC analysis demonstrated HV antigens and EM analysis identified numerous intranuclear HV particles in this case study whale [7].

HV can be ubiquitous and its capacity to cause disease in cetaceans is unclear and may be varied depending upon an individual’s condition and species type [7-10]. Recently, systemic herpes viral lesions have been reported in a striped dolphin, probably secondary to immunosuppression caused by morbillivirus co-infection [11].

Tissues from the adult old beaked whale in this study were negative for morbillivirus infection but it had a poor body condition, empty stomach containing small foreign bodies, multiorgan parasitic infestation, chronic adrenalitis and membranous glomerulonephritis. One or more of these conditions/lesions have been described in immunocompromised hosts [23].

Therefore, what is the explanation for an acute viral infection in the tubuloepithelial renal cells associated with chronic lymphoproliferative interstitial nephritis? In dogs, infectious viral canine hepatitis is a well-documented mechanism of interstitial nephritis. Following recovery of dogs from the acute phase and the loss of virus from systemic circulation, virus only reappears in tubular epithelial cells. Thus, virus can persist in the tubular epithelium and cause necrosis as a result of viral-induced cytolysis. This process is followed by lymphoplasmacytic interstitial nephritis [23].

Of particular interest are viral infections in the kidney of immunocompromised human hosts. Epstein Barr virus (EBV) is a ubiquitous HV that can persist in a latent form. B lymphocytes are the usual site of latent infection, whereas epithelial cells usually contain active virus. EBV infection of renal proximal tubular cells may induce cellular immune responses that result in damage to renal interstitium causing interstitial nephritis [24]. The beaked whale in this study presents similarities with viral infections in dogs and humans and provokes questions regarding the prevalence of viral latent infection in cetaceans.

The novel sequence obtained from the kidney of the beaked whale is divergent from previous HV sequences obtained from relative host species of the *Ziphiid* family:

From two γ-HV sequences belonging to the tentatively named *Ziphiid herpesvirus 1* (ZiHV-1) [19] found in two *M. densirostris* stranded on the Atlantic coast of the USA (GenBank accession numbers AY803337 and AY949828) [8,9], where both sequences were 100% identical in a 47 aa region in this study.

From the alpha-HV sequence found in a *M. densirostris* stranded in the eastern Atlantic in 2005 (GenBank accession number GU066291) [7]. As it is unclear whether this sequence belongs to an alpha-HV host specific to the *Ziphiid* family or to the *Delphinidae* family, and because almost identical sequences were found in two *S. coeruleoalba* (*Delphinidae* family) from the Mediterranean sea (GenBank accession numbers GU068981 and HQ214675) [11,18], no putative virus name has been proposed to date for this α-HV.

From one γ-HV sequence detected in a *Mesoplodon stejnegeri* stranded in the Sea of Japan [20] whose putative name, following nomenclature suggested by Maness et al., is “*Ziphiid herpesvirus 2*” (ZiHV-2).

Thus, following Maness et al., suggested nomenclature, the putative name “*Ziphiid herpesvirus 3*” (ZiHV-3) has been proposed for the putative novel virus sequence identified in this study (GenBank accession number JN863234).

## Conclusions

Kidney lesions and virus-associated infection have rarely been described in cetaceans. Therefore, data from this case study suggests that the identification of HV infection can be used for the etiological diagnosis of tubulointerstitial and lymphoplasmacytic interstitial nephritis in beaked whales. This conclusion highlights the need for further investigation of other stranded cetaceans that combines pathological and molecular analytical studies. These studies can contribute to a better understanding of HV infection and disease, using cetaceans as a marine mammal model.

## Competing interests

Authors declare that they have no competing interests.

## Authors’ contributions

All authors read and approved the final manuscript.

## References

[B1] Barr B Dunn JL Daniel MD Banford A Herpes-like viral dermatitis in a beluga whale (*Delphinapferus leucas*) J Wildl Dis 1989 25 608 611 2554002 10.7589/0090-3558-25.4.608

[B2] Martineau D Lagaci A Beland P Higgins R Armstrong D Shugart LR Pathology of stranded beluga whales (*Delphinapterus leucas*) from the St. Lawrence estuary, Quebec, Canada J Comp Pathol 1988 98 287 311 10.1016/0021-9975(88)90038-2 3134469

[B3] Kennedy S Lindstedt IJ McAliskey MM McConnell SA McCullough SJ Herpesviral encephalitis in a harbor porpoise (*Phocoena phocoena*) J Zoo Wildl Med 1992 23 374 379

[B4] Van Bressem MF Van Waerebeek K Garcia-Godos A Dekegel D Pastoret PP Herpes-like virus in dusky dolphins, *Lagenorhynchus obscurus*, from coastal Peru Mar Mamm Sc 1994 10 354 359 10.1111/j.1748-7692.1994.tb00490.x

[B5] Mikaelian I Tremblay MP Montpetit C Tessaro SV Cho HJ House C Measures L Martineau D Seroprevalence of selected viral infections In a population of beluga whales (*Delphinapterus leucas*) in Canada Vet Rec 1999 144 50 51 10.1136/vr.144.2.50 10028586

[B6] Blanchard TW Santiago NT Lipscomb TP Garber RL McFee WE Knowles S Two novel alphaherpesviruses associated with fatal disseminated infections in Atlantic bottlenose dolphins J Wildl Dis 2001 37 297 305 11310880 10.7589/0090-3558-37.2.297

[B7] Arbelo M Sierra E Esperón F Watanabe TT Bellière EN Espinosa De Los Monteros A Fernández A Herpesvirus infection with severe lymphoid necrosis affecting a beaked whale stranded in the Canary Islands Dis Aquat Organ 2010 89 261 264 20481092 10.3354/dao02208

[B8] Smolarek Benson KA Manire CA Ewing RY Saliki JT Townsend FI Ehlers B Romero CH Identification of novel alpha and gammaherpesviruses from cutaneous and mucosal lesions of dolphins and whales J Virol Methods 2006 136 261 266 10.1016/j.jviromet.2006.03.033 16784784

[B9] Saliki JT Cooper EJ Rotstein DS Caseltine SL Pabst DA McLellan WA Govett P Harms C Smolarek KA Romero CH A novel gammaherpesvirus associated with genital lesions in a Blainville’s beaked whale (*Mesoplodon densirostris*) J Wildl Dis 2006 42 142 148 16699156 10.7589/0090-3558-42.1.142

[B10] van Elk CE van de Bildt MWG de Jong AAW Osterhaus ADME Kuiken T Genital herpesvirus in bottlenose dolphins (*Tursiops truncatus*): cultivation, epidemiology, and associated pathology J Wildl Dis 2009 45 895 906 19901366 10.7589/0090-3558-45.4.895

[B11] Soto S Gonzalez B Willoughby K Maley M Olvera A Kennedy S Marco A Domingo M Systemic herpesvirus and morbillivirus Co-infection in a striped dolphin (*stenella coeruleoalba*) J Comp Pathol 2012 146 269 273 10.1016/j.jcpa.2011.04.002 21601871

[B12] Kelly CJ Neilson EG Brenner B, Rector F Tubulointerstitial diseases The kidney 1996 Philadelphia, PA: W.B. Saunders Co 1655 1679

[B13] Fernández A Edwards JF Rodríguez F Espinosa De Los Monteros A Herráez P Castro P Jaber JR Martín V Arbelo M Gas and fat embolic syndrome involving a mass stranding of beaked whales (family ziphiidae) exposed to anthropogenic sonar signals Vet Pathol 2005 42 446 457 10.1354/vp.42-4-446 16006604

[B14] Fernández A Esperón F Herraéz P De Los Monteros AE Clavel C Bernabé A Sánchez-Vizcaino JM Verborgh P DeStephanis R Toledano F Bayón A Morbillivirus and pilot whale deaths, Mediterranean Sea Emerg Infect Dis 2008 14 792 794 10.3201/eid1405.070948 18439363 PMC2600256

[B15] Esperon F Fernandez A Sanchez-Vizcaino JM Herpes simplex-like infection in a bottlenose dolphin stranded in the Canary Island Dis Aquat Organ 2008 81 73 76 18828564 10.3354/dao01915

[B16] VanDevanter DR Warrener P Bennett L Schultz ER Coulter S Garber RL Rose TM Detection and analysis of diverse herpesviral species by consensus PCR J Clin Microbiol 1996 34 1666 1671 8784566 10.1128/jcm.34.7.1666-1671.1996 PMC229091

[B17] Manire CA Smolarek KA Romero CH Kinsel MJ Clauss TM Byrd L Proliferative dermatitis associated with a novel alphaherpesvirus in an Atlantic bottlenose dolphin (*Tursiops truncatus*) J Zoo Wildl Med 2006 37 174 181 10.1638/05-006.1 17312797

[B18] Bellière EN Esperón F Arbelo M Muñoz MJ Fernández A Sánchez-Vizcaíno JM Presence of herpesvirus in striped dolphins stranded during the cetacean morbillivirus epizootic along the Mediterranean Spanish coast in 2007 Arch Virol 2010 155 1307 1311 10.1007/s00705-010-0697-x 20495987

[B19] Maness HT Nollens HH Jensen ED Goldstein T LaMere S Childress A Sykes J St Leger J Lacave G Latson FE Wellehan JF Jr Phylogenetic analysis of marine mammal herpesviruses Vet Microbiol 2011 149 23 29 10.1016/j.vetmic.2010.09.035 21055885

[B20] Miyoshi K Nishida S Sone E Tajima Y Makara M Yoshioka M Nakamura M Yamada TK Koike H Molecular identification of novel alpha- and gammaherpesviruses from cetaceans stranded on Japanese coasts Zoolog Sci 2011 28 126 133 10.2108/ZSJ.28.126 21303205

[B21] McGeoch DJ Rixon FJ Davison AJ Topics in herpesvirus genomics and evolution Virus Res 2006 117 90 104 10.1016/j.virusres.2006.01.002 16490275

[B22] International committee on taxonomy of viruses, UK (ICTV) 2002 [http://www.ictvdb.rothamsted.ac.uk/Ictv/index.htm]

[B23] Confer A Panciera RJ McGavin MD, Carlton WW, Zachary JF The urinary system Thomson’s special veterinary pathology 2001 3 Mosby. St: Louis. Missouri 235 278

[B24] Becker JL Miller F Nuovo GJ Josepovitz C Schubach WH Nord EP Epstein-Barr virus infection of renal proximal tubule cells: possible role in chronic interstitial nephritis J Clin Invest 1999 104 1673 1681 10.1172/JCI7286 10606621 PMC409878

